# Alzheimer’s disease pathogenetic progression is associated with changes in regulated retained introns and editing of circular RNAs

**DOI:** 10.3389/fnmol.2023.1141079

**Published:** 2023-05-05

**Authors:** Karol Andrea Arizaca Maquera, Justin Ralph Welden, Giorgi Margvelani, Sandra C. Miranda Sardón, Samantha Hart, Noémie Robil, Alvaro Gonzalo Hernandez, Pierre de la Grange, Peter T. Nelson, Stefan Stamm

**Affiliations:** ^1^Department of Molecular and Cellular Biochemistry, University of Kentucky, Lexington, KY, United States; ^2^Sanders-Brown Center on Aging, University of Kentucky, Lexington, KY, United States; ^3^GenoSplice, Paris, France; ^4^DNA Services Facility, University of Illinois, Urbana, IL, United States; ^5^Alzheimer’s Disease Research Center and Department of Pathology and Laboratory Medicine, University of Kentucky, Lexington, KY, United States

**Keywords:** Alzheimer, Braak stage, circular RNAs, alternative splicing, gene expression, retained intron

## Abstract

**Introduction:**

The molecular changes leading to Alzheimer’s disease (AD) progression are poorly understood. A decisive factor in the disease occurs when neurofibrillary tangles (NFT) composed of microtubule associated protein tau (MAPT) form in the entorhinal cortex and then spread throughout the brain.

**Methods:**

We therefore determined mRNA and circular RNA changes during AD progression, comparing Braak NFT stages I-VI. Total RNA was isolated from human brain (entorhinal and frontotemporal cortex). Poly(A)+ RNA was subjected to Nanopore sequencing, and total RNA was analyzed by standard Illumina sequencing. Circular RNAs were sequenced from RNase R treated and rRNA depleted total RNA. The sequences were analyzed using different bioinformatic tools, and expression constructs for circRNAs were analyzed in transfection experiments.

**Results:**

We detected 11,873 circRNAs of which 276 correlated with Braak NFT stages. Adenosine to inosine RNA editing increased about threefold in circRNAs during AD progression. Importantly, this correlation cannot be detected with mRNAs. CircMAN2A1 expression correlated with AD progression and transfection experiments indicated that RNA editing promoted its translation using start codons out of frame with linear mRNAs, which generates novel proteins.

**Discussion:**

Thus, we identified novel regulated retained introns that correlate with NFT Braak stages and provide evidence for a role of translated circRNAs in AD development.

## Introduction

Alzheimer’s disease (AD) is characterized by the occurrence of beta amyloid plaques, tau protein inclusions, and neurodegeneration (Jack et al., 2016, 2018). A decisive molecular event for AD progression is the aggregation of hyperphosphorylated tau proteins into neurofibrillary tangles (NFT) that affects the entorhinal cortex early in the disease course, and then in more advanced cases NFTs are seen throughout the brain. The anatomical location of NFTs is used to classify AD into six stages as identified by Braak and Braak (1991, 1995), where NFTs in stage I are localized to the entorhinal cortex before they spread to the neocortex in stage VI (Braak and Braak, 1991, 1995). The stage of tau pathology correlates strongly with cognitive impairment (Giannakopoulos et al., 2003; Nelson et al., 2012). The genetic substrates for tau pathology origination and propagation are incompletely characterized to date.

Numerous genes have been associated with AD. One of best documented AD risk genes is apolipoprotein E (ApoE), a protein that binds and transports lipids (Strittmatter et al., 1993), and also likely binds to beta amyloid plaques (Namba et al., 1991; Jiang et al., 2008) which possibly exerts an immune response (Gale et al., 2014). It is well documented that reactive microglia is associated with AD and several genes involved in the immune response have been identified as risk genes, among them TREM2, CR1, SHIP1, BIN1, CD33, PICALM, CLU, PCLG2, Clec7A, and MS4A (Gale et al., 2014; Karch and Goate, 2015). A genome wide association study identified 75 risk loci that were associated with beta amyloid, tau and reactive microglia, supporting their involvement in AD (Bellenguez et al., 2020, 2022).

Transcriptome analysis using human *post mortem* tissue including single cell RNAseq revealed hundreds of changes in gene expression (reviewed in Bagyinszky et al., 2020), as well as numerous changes in alternative splicing that suggests that the effect of PICALM, CLU, and PTK2B is mediated through changes in pre-mRNA splicing (Raj et al., 2018).

Circular RNAs (circRNAs) are a recently identified class of RNAs, generated through backsplicing of a 5′ splice site to a downstream 3′ splice site (Yang et al., 2022). Changes in circular RNA expression have previously been detected in parietal cortex in AD cases (Dube et al., 2019). Collectively, the expression changes of ten circRNAs have a high predictive value for the cognitive impairment. The association of circular RNA expression with AD has also been found in other brain regions (Lo et al., 2020; Cochran et al., 2021). The circular RNAs from two AD-associated genes, presenelin 1 (PSEN1) and tau have been studied in detail. circPSEN1 increases during autosomal-dominant Alzheimer’s disease progression (Chen et al., 2022). After undergoing RNA editing, circular RNAs from the tau pre-mRNA encompassing the microtubule binding sites are translated into proteins that promote tau aggregation *in vitro* (Welden et al., 2022).

Here we analyze the transcriptome during progression of AD using postmortem samples of entorhinal and temporal cortex with Braak stages I–VI. We confirmed earlier studies showing large changes in transcription levels and alternative splicing that in part reflect the changes in cellular content. We concentrated on regulated retained introns (Hart et al., 2022) that have not been studied in human systems and found them to be deregulated in AD. Finally, we could confirm changes in circular RNA expression and found that collectively their A > I RNA editing is upregulated during AD progression in entorhinal cortex. Transfection experiments indicate that similar to MAPT (Welden et al., 2022), circMAN2A1, a highly expressed circRNA that correlates with AD progression, can be translated after undergoing RNA editing. The data suggest that during AD progression circRNAs could act on the protein level after their translation is activated through A > I RNA editing.

## Materials and methods

### RNA isolation

Total RNA was isolated using the RNeasy Mini Kit (Qiagen, Hilden, Germany). Total polyA+ RNA was selected for Nanopore sequencing using Dynabeads™ mRNA Purification Kit (Invitrogen, MA).

### Nanopore sequencing

Prior to preparing the poly (A+) RNA for Nanopore sequencing, the concentration of the sample was determined by Qubit (Invitrogen, CA). cDNA for sequencing was prepared using the Direct RNA Sequencing Kit (Oxford Nanopore Technologies, Oxford, United Kingdom) using FLO-MIN106D flow cells. The MinION cells were run for 72 h at 180 volts for each sample, and a new flow cell was used for each sample. Base-calling was done after the runs were completed.

### Nanopore data analysis for retained intron validation

Base-calling was performed using Guppy (Oxford Nanopore Technologies). Read correction using Illumina samples was performed using TALC. Quality control of data was performed using FastQC, Samtools, RSeQC, and NanoPlot (De Coster et al., 2018). Reads were aligned using Minimap2 (Li, 2018). Validation of retained introns was performed using custom Perl scripts.

### RNAseq

Total RNAs were run on a Fragment Analyzer (Agilent, CA) to evaluate RNA integrity. RNAseq libraries were constructed with the TruSeq Stranded mRNA Sample Prep kit (Illumina, CA). Briefly, polyadenylated messenger RNAs (mRNAs) were enriched from 500 ng of high-quality DNA-free total RNA with oligodT beads. The mRNAs were chemically fragmented, annealed with a random hexamer and converted to double stranded cDNAs, which were subsequently blunt-ended, 3′-end A-tailed and ligated to indexed adaptors. Each library was ligated to a uniquely dual indexed adaptor (unique dual indexes) to prevent index switching. The adaptor-ligated double-stranded cDNAs were amplified by PCR for eight cycles with the Kapa HiFi polymerase (Roche, CA) to reduce the likeliness of multiple identical reads due to preferential amplification. The final libraries were quantified with Qubit (ThermoFisher, MA) and the average library fragment length was determined on a Fragment Analyzer. The libraries were diluted to 10 nM and further quantitated by qPCR on a CFX Connect Real-Time qPCR system (Biorad, Hercules, CA) for accurate pooling of the barcoded libraries and maximization of number of clusters in the flow cell.

The libraries were sequenced from both ends of the fragments for a total of 150 nt from each end in a NovaSeq SP flow-cell (Illumina). The fastq read files were generated and demultiplexed with the bcl2fastq v2.20 Conversion Software (Illumina, San Diego, CA). The quality of the demultiplexed fastq files was evaluated with the FastQC software, which generates reports with the quality scores, base composition, k-mer, GC and N contents, sequence duplication levels and overrepresented sequences.

### RNAseq of circRNAs

Construction of libraries and sequencing on the Illumina NovaSeq 6000 were performed at the Roy J. Carver Biotechnology Center at the University of Illinois at Urbana-Champaign. Ribosomal RNA was removed using the Ribo minus kit (Thermo). After purification with RNA cleanup columns (Zymo Research), the RNAs were incubated with RNase R (NEB) at 37°C for 15 min, followed by purification with RNA cleanup columns. Eluted RNAs were converted into RNAseq libraries with the TruSeq Stranded Total RNA kit (Illumina), starting at the fragmentation step. Briefly, the circular RNAs were chemically fragmented, annealed with a random hexamer and converted to double stranded cDNAs, which were subsequently blunt-ended, 3′-end A-tailed and ligated to indexed adaptors. Each library was ligated to a uniquely dual indexed adaptor (unique dual indexes) to prevent index switching. The adaptor-ligated double-stranded cDNAs were amplified by PCR for eight cycles with the Kapa HiFi polymerase (Roche, CA) to reduce the likeliness of multiple identical reads due to preferential amplification. The final libraries were quantitated with Qubit (ThermoFisher, MA) and the average library fragment length was determined on a Fragment Analyzer. The libraries were diluted to 10 nM and further quantitated by qPCR on a CFX Connect Real-Time qPCR system (Biorad, Hercules, CA) for accurate pooling of the barcoded libraries and maximization of number of clusters in the flowcell.

### CircRNA identification

In the sequencing, data quality, reads repartition (e.g., for potential ribosomal contamination), and insert size estimation were performed using FastQC, Picard-Tools, Samtools and rseqc for both type of samples. For circRNA samples, we used CIRI-full (Zheng et al., 2019) to detect circRNA and reconstruct full-lenght isoform, CIRIquant (Zhang et al., 2020) is used for quantification and differential analysis. In CIRI pipeline, all reads are aligned on the hg19 Human genome assembly and Ensembl 82 annotations are used for genes definition.Clusterings are performed using using “dist” and “hclust” functions in R, using Euclidean distance and Ward agglomeration method. Correlation with Braak score was performed using spearman correlation.

## Results

### Changes in mRNA expression and alternative splicing during AD progression in entorhinal and temporal cortex

#### Significant changes in cell composition during AD progression in temporal and entorhinal cortex

In order to determine changes in RNA expression during AD progression, we determined gene expression in the entorhinal and temporal cortex from seven subjects with Braak NFT stages I–VI and control (Supplementary Table 1). All post-mortem intervals for included tissue was <5 h. Tissue was dissected at brain autopsy and snap-frozen in liquid nitrogen, then stored at −80°C until use. The temporal cortex was manually dissected as it was thawed into white and gray matter, whereas the entorhinal cortex was not dissected in this manner because some of the gray-white junctions were more indistinct. Braak NFT stages were assessed as described previously at the University of Kentucky AD Research Center (Braak et al., 2006; Smith et al., 2018).

RNA from these samples was analyzed using standard Illumina RNAseq using paired-end reads of 150 bp length. We obtained between 108 and 160 million read pairs per sample, from which 90–93% could be uniquely mapped. On average the samples expressed 17,528 genes (Supplementary Table 1). The dataset contained between 55 and 89 million exon-exon junctions and 2.6 billion reads. Similar to other datasets (Hart et al., 2022), mitochondrial genes showed highest expression, MT-CO1/2/3 // ND3/4/5/6 representing 5.67%, MT-RNR1 1.32% and MT-ND1/2 0.92% of all mRNAs. The highest expressed non-mitochondrial gene was myelin basic proteins (MBP) representing 1.85% of all reads.

Using unsupervised clustering, we found that the samples cluster according to Braak stages, which was most pronounced in entorhinal cortex (Supplementary Figure 1).

#### RNA signatures indicate a change in cell composition during AD progression in temporal and entorhinal cortex

Changes in gene expression could be caused by disease-associated changes in a single cell or by a change in cellular composition. We used cell-type specific RNA signatures (Zhang et al., 2016) to address this question and saw a decrease of neuronally expressed RNAs and an increase of endothelial cell expressed RNA in entorhinal cortex. Although showing the same trend, this correlation did not reach statistical significance in temporal cortex (Supplementary Figure 2).

**Figure 1:**
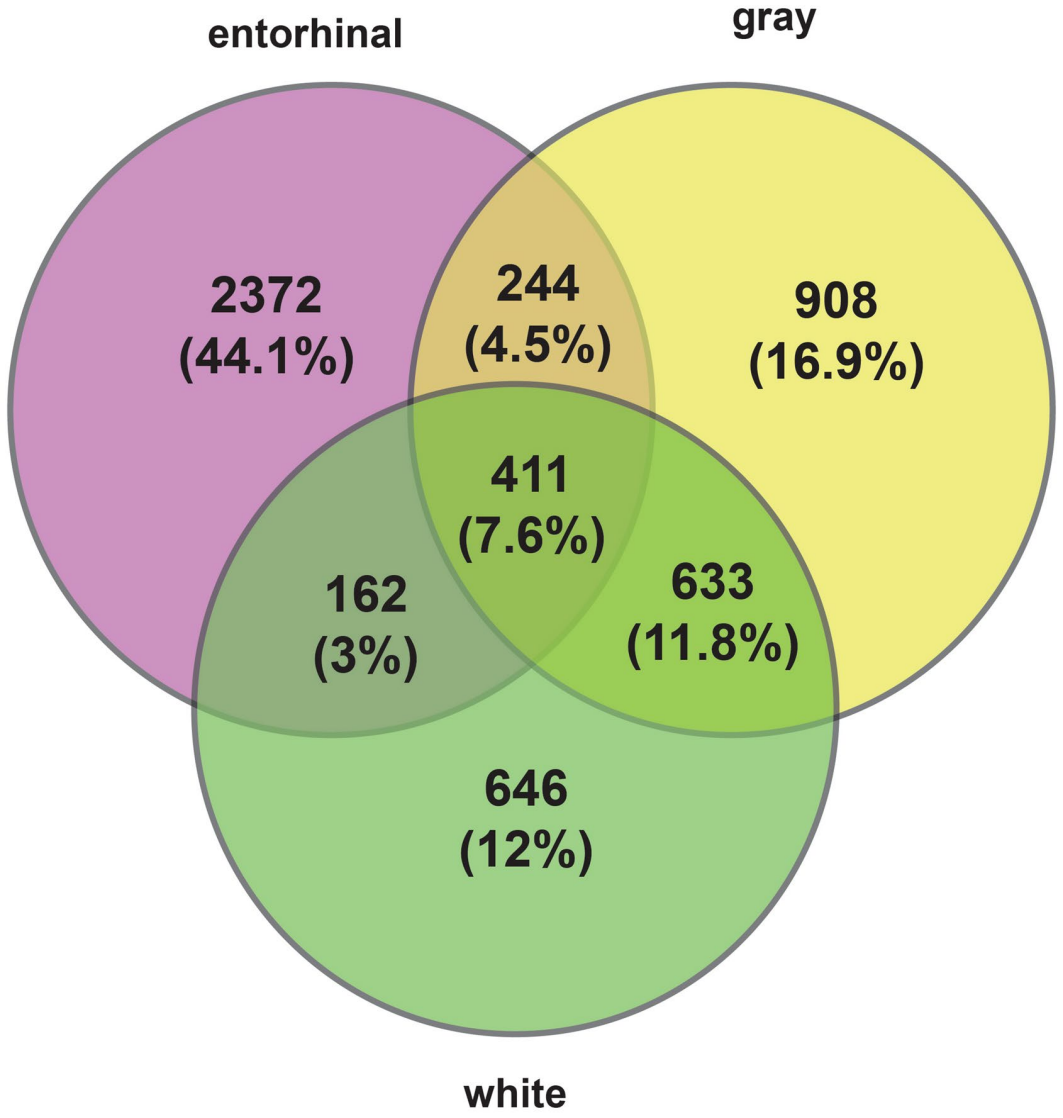
Changes in gene expression in entorhinal cortex, temporal cortex white and gray matter.

Comparing the combined Braak stages 1 + 2 with 5 + 6, we identified 3,189 changes in gene expression between high and low Braak stages in entorhinal cortex (Supplementary Table 2); 2,196 changes in gray matter of temporal cortex (Supplementary Table 3), and 1,852 changes in white matter (Supplementary Table 4). A total of 411 genes changed their expression in all tissues during changes in AD progression (Figure 1). The strongest changes in gene expression are listed in Table 1A.

**Table 1:** Overview of changes in gene expression.

(A) Genes with the highest changes in expression						
	Entorhinal		Gray		White	
Number of expressed genes	18,291		17,692		17,872	
# regulated genes (up/down)	3,189 (1,547/1,642)		2,196 (1,109/1,087)		1,852 (697/1,155)	
Top 5 up-and down-regulated annotated genes	HLA-DRB5	OPN	HLA-DRB5	HBG2	HLA-DRB5	CT45A10
	CCL2	CT45A10	FCGR2B	EFHB	FOSL1	HBG2
	CD44	MIR378I	CXCR4	TPSB2	FCGR2B	EFHB
	SERPINA3	EFHB	OSGIN1	METTL27	OSGIN1	NPY2R
	NGFR	FNDC1	RGS1	SLC22A6	MAFF	METTL27
**(B) Genes previously associated with AD found in our dataset**						
	**Entorhinal**		**Gray**		**White**	
	8 genes		12 genes		6 genes	
Up-regulated genes	ERBIN, MAP7,**MAN2A1**,**DOCK1**, BIN1, RIN3, PDE4B, PICALM		CHRNA5, HTR1B, PLEKHM3, PHC3, APPL1, APPBP2, CD2AP, CORO1C, LINC-PINT, SLAIN2, MAPT, RABEP1		CNTNAP2, **MAPT** , PHC3, SLAIN2, CORO1C, LINC-PINT	
	23 genes		3 genes		4 genes	
Down-regulated genes	GRIN3A, HTR3A, CHRNB2, HTR1A, CX3CR1, CHRM1, ICA1, CHRNA7, GRIA2, GRIN1, TSPOAP1, PTK2B, CHRM3, PSEN2, KCNN2, PLD3, ACE, CDK5, NLRP3, RNASEH2B, WDR78, ADGRB3, MEG3		CX3CR1, GRIN2C, BCKDK		CX3CR1, ADAMTS4, ECHDC3,**PSEN1**	

Bold indicated genes also strongly express circRNAs, some of which change their expression during AD progression (Table 3). Underlined genes are deregulated in more than one experimental condition.

We found a considerable overlap with our sequencing data and the results of previous sequencing projects (Bellenguez et al., 2020; Meng et al., 2020; Tábuas-Pereira et al., 2020; Wang M. et al., 2021; Table 1B), further validating our dataset. Importantly, changes in gene expression correlate in part with alternations of the in cellular tissue composition, most striking with the relative loss of neurons.

#### Changes in alternative splicing reflect Braak stages

**Figure 2:**
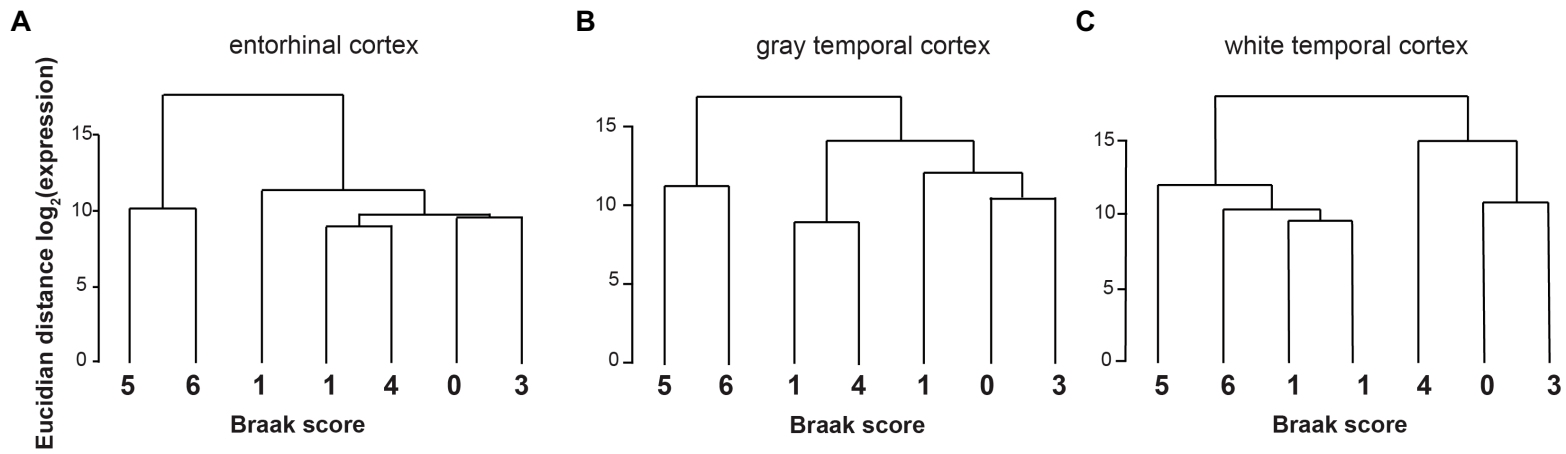
Cluster analysis from PSI and Braak stages. The Euclidian distance in splice site usage, measured as log_2_(changes in per cent spliced in) was calculated for each Braak stage. **(A)** Entorhinal cortex, **(B)** gray temporal cortex, and **(C)** white temporal cortex.

So far, most studies on gene expression in Alzheimer’s disease focused on mRNA expression changes (Raj et al., 2018). We therefore investigated in detail the changes in alternative splicing in our dataset. Using exon junction ratios, we calculated the exon-inclusion ratio [percent spliced in (PSI)], defined as the ratio of the expression of all isoforms containing a certain exon over the expression of all isoforms of this gene. In entorhinal cortex, 3,115 splicing events in 2,146 genes are alternatively regulated, that include 349 alternative first exons, which could be regulated by transcriptional start site selection (Supplementary Table 2). Similarly, in temporal cortex (Supplementary Table 5) we observed 2,757 differentially regulated splicing events, including 292 alternative first exons. We next performed cluster analysis of the PSI values and Braak stages and observed a strong correlation between changes in alternative splicing and Braak stages (Figure 2).

We next concentrated on two novel RNA processing events: the regulated usage of retained introns and the expression of circular RNAs.

### Regulated retained introns are present in human brain and change during AD progression

Differentially regulated intron retention has been observed during mouse germ cell differentiation (Naro et al., 2017) and in a stimulation paradigm of cultured mouse neurons (Mauger et al., 2016). Recently, we identified retained introns in polyadenylated pre-mRNA in a rat model of spinal cord injury (Hart et al., 2022). Stimulation studies in mouse cell culture indicate that retaining introns in nuclear poly (A+) RNA allows cells to rapidly respond to stimuli by removing one intron, which is faster than processing numerous introns from a pre-mRNA (Mauger et al., 2016).

#### Identification of regulated retained introns in human brain samples

**Figure 3:**
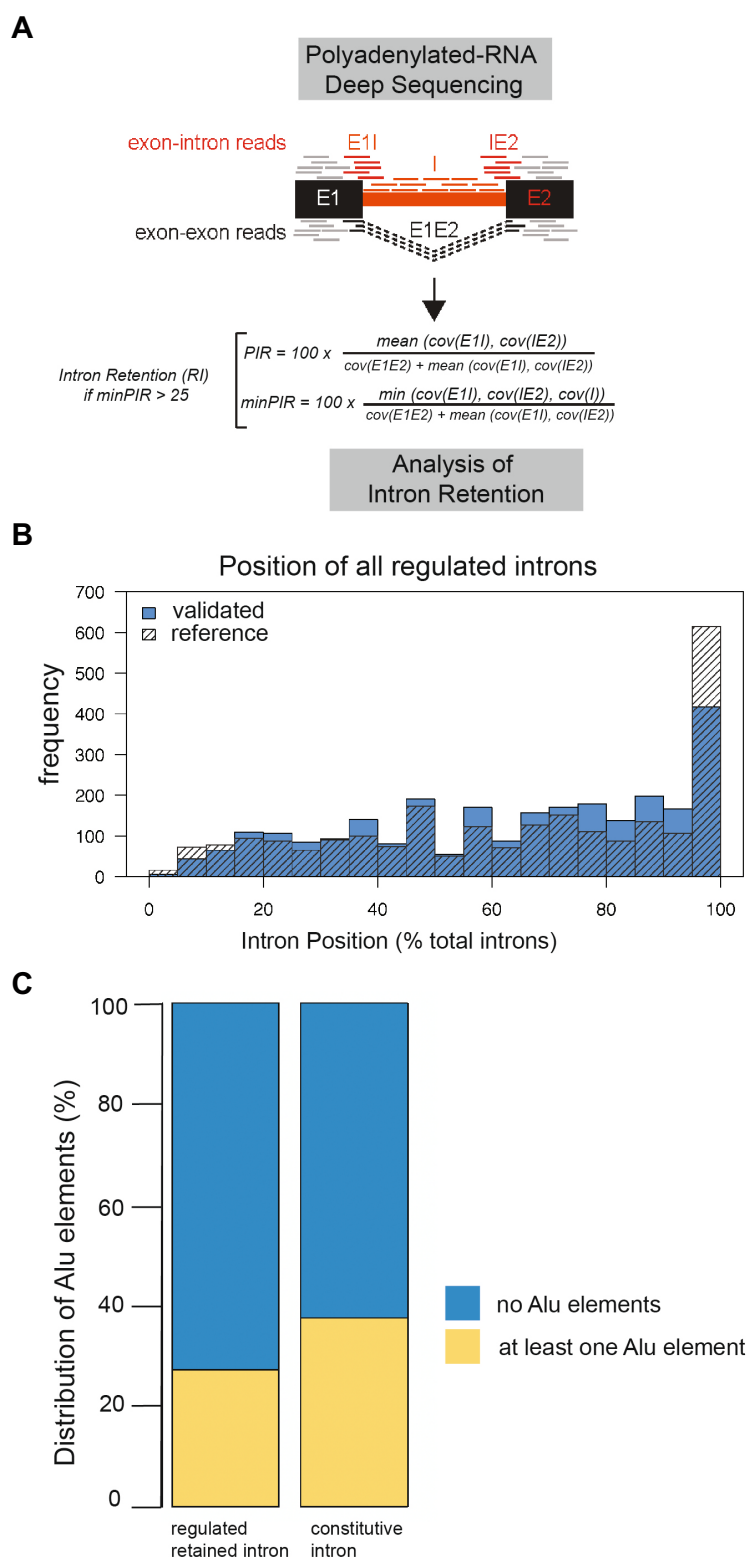
Properties of human regulated retained introns. **(A)** Algorithm to identify regulated retained introns from Illumina RNAseq data. **(B)** Position of the regulated retained introns in pre-mRNA. All mRNAs were normalized to a length of 100 and the position of each regulated retained intron tabulated. **(C)** Per cent of introns containing at least one Alu-element in regulated retained introns compared to control. The difference is significant, Chi2 *p*−value = 7.79e^−207^.

To investigate whether regulated retained introns exist in human brain, we determined their presence in our dataset, using a previously established bioinformatic pipeline (Mauger et al., 2016). Briefly, we detected retained introns in RNA-Seq data by combining exon-intron junction reads flanking an intron with reads covering the body of the intron (Figure 3A). We detected around 10,000 retained introns per sample, which corresponds to 0.51–0.63 introns per expressed gene in a given sample.

Using Sanger sequencing it is technically not possible to discriminate intron retention in mRNA from genomic DNA contamination or pre-mRNA, as both molecules have the same sequence. As RNA-Seq reads are only 150 nt long, the structure of individual molecules is interpolated through sequence alignment, i.e., the connectivity of exons in a given molecule is not clear. We thus employed nanopore sequencing that detects polyadenylated RNA, due to the priming of the polyA-tail with oligo dT linked to nanopore adaptors. After cDNA synthesis, nanopore sequencing detects the RNA of a single RNA-cDNA hybrid transported through the nanopore and thus directly indicates the exon-intron structure (Wang Y. et al., 2021). We considered retained introns to be validated through Nanopore sequencing if they occurred in more than 10 nanopore reads. This cutoff considered only sufficiently expressed transcripts and assured accurate mapping.

Using these criteria, we confirmed 2,655 retained introns using Nanopore sequencing (Supplementary Table 6).

Thus, similar to rat spinal cord and mouse neuronal cultures, human brain expresses polyadenylated RNAs that contains regulated retained introns.

#### General properties of retained introns in human brain

We next characterized the human retained introns by determining their location, Alu-element content, size and splice site qualities. The introns are found throughout the pre-mRNA, indicating that most retained introns would introduce premature stop codons if translation occurs, effectively switching off genes (Figure 3B). Next, we asked whether regulated retained introns contain more Alu-elements and searched for Alu-element sequences defined from the UCSC repeat masker in our dataset. 6,553 (27%) regulated retained introns contain at least one Alu-elements, whereas 17,509 retained introns lack them. This compared to 54,439 (37%) of control introns containing at least one Alu-element, whereas 90,764 lack Alu-elements (Figure 3C).

**Figure 4:**
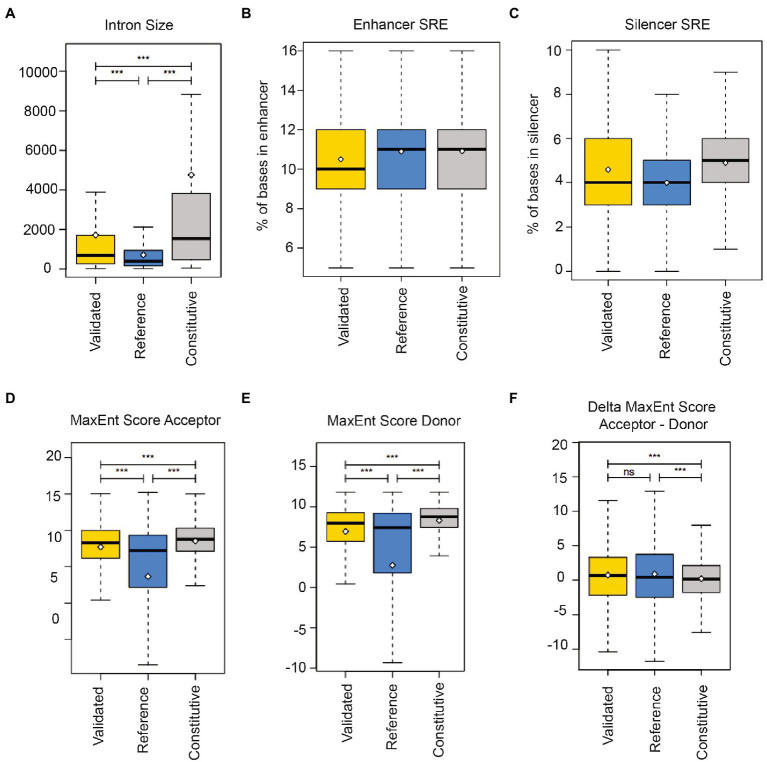
Regulatory elements in human regulated retained introns. **(A)** Comparison of regulated retained intron length that were validated by Nanopore sequencing (validated) with regulated retained introns determined by RNAseq (reference) and constitutively spliced introns (constitutive). **(B)** Occurrence of enhancer splicing regulatory elements (SRE) in introns. The box plots show the proportion of intron bases that overlap with enhancers. **(C)** Occurrence of silencer splicing regulatory elements (SRE) in introns. The box plots show the proportion of intron bases that overlap with silencers. **(D)** 3′ splice site quality of regulated retained introns and **(E)** 5′ splice site quality of regulated retained introns, **(F)** Combined splice site quality of regulated retained introns.

We compared nanopore-validated introns with all known human retained introns derived from our dataset and the FastDB annotations (de la Grange et al., 2007), as well as constitutively spliced introns from our dataset. The nanopore-sequencing validated retained introns are significantly shorter (mean 1716 ± 4,221 bp) than constitutively spliced introns (mean 4,763 ± 14,586 bp; Figure 4A), indicating a selection of shorter introns.

To determine regulatory splicing elements, we used enhancer and silencer elements compiled in Sroogle (Schwartz et al., 2009). Enhancer sequences were from Fairbrother et al. (2002), from ESEfinder (Cartegni et al., 2003) and from Zhang and Chasin (2004) where we used elements with a score equal or >0.8. This resulted in a list of 1,471 distinct enhancer sequences (6–8 bp). Silencer sequences were from Wang et al. (2004) and from Zhang and Chasin (2004) with a score equal or >0.8, resulting in 307 distinct silencer sequences (6 or 8 bp). We then computed the percentage of bases overlapping with at least one splicing regulatory element. We found that retained introns and their flanking exons (Figure 3A) contain less enhancer sequences compared to reference (Figure 4B). Similarly, regulated retained introns identified by us and found in databases contain less silencer sequences than constitutively spliced introns (Figure 4C).

The regulated retained introns are flanked by 3′ and 5′ splice sites that are weaker than the ones of constitutive introns (Figures 4D,E). However, when the strength of the intron-flanking splice sites is combined, there is no difference to constitutive spliced introns (Figure 4F). This suggests that the regulation of intron retention occurs at one of the two splice sites and possibly involves the recognition of the flanking exons, rather than only relying on intronic sequences.

#### Conservation of regulated intron retention in mouse, rat, and human

We next compared our datasets of retained introns from mouse, rat and human systems. We used blastn (Altschul et al., 1990) to detect the reciprocal best hits (RBHs) between exons of a pair of organisms, i.e., human vs. mouse and human vs. rat (hg19/mm10 and hg19/rn6). A pair of RBH exons was considered as orthologs when they showed more than 70% sequence identity and more than 50% coverage. A pair of introns was considered orthologous when both flanking RBH exons were found orthologous using these criteria. In order to obtain a list of conserved regulated retained introns, we compared the list of 24,062 retained introns from RNA-Seq of human samples with all known conserved introns in human and mouse (16,313 introns, hg19/mm10), and 13,138 introns conserved between human and rat (hg19/rn6). We then compared this dataset with previously identified regulated retained introns from mouse neuronal cultures (Mauger et al., 2016) and 2,354 regulated retained introns in identified in rat (Hart et al., 2022). This analysis identified 13 regulated retained introns conserved in mouse and human and 100 regulated introns conserved between rat spinal cord and human brain. Three of these retained introns were conserved in all three species, MAT2A (intron 10), MAX (intron 6) and NEXMIF (intron 4). The conserved regulated retained introns are shown in Supplementary Table 7.

The regulated retained introns from three species show common features: they are shorter than non-regulated introns, and their hosting genes are longer than average and have more introns (Table 2).

**Table 2:** Properties of conserved regulated introns. Mean length from FAStDB of the introns is indicated.

	Intron length (nt)	Gene length (nt)	Introns number	Relative intron position
conserved regulated introns	1,770	116,336	17.04	0.672
reference introns	6,429	59,655	9.15	0.745

Gene ontology (WebGestaltR; Liao et al., 2019) analysis showed regulation of membrane potential, ion transmembrane transport and glutamate receptor signaling pathways as the top functional categories. Of note, all four AMPA glutamate receptors GRIA1-4 exhibit a retained regulated intron. In the case of GRIA1, this regulated retained intron is located between the regulated ‘flip-flop’ alternative exons that regulate desensitization kinetics of the receptor (Mosbacher et al., 1994). This intron was validated using nanopore sequencing.

Five of these conserved regulated retained introns were in splicing factors: SFSWAP, SRRM1, CLK4, hnRNPL, and PNISR. We found that introns 1, 2, 14, and 15 of the human SFSWAP were regulated retained introns, included in most samples. However, there is a negative correlation (−0.83) between SFSWAP intron 2 and SWAP intron 15 and Braak stages in entorhinal cortex (expression change −10.75%). Of note, intron 2 was shown to be autoregulated by SWAP protein (Sarkissian et al., 1996). CLK1 autoregulates protein expression by regulating exon 4 and intron 4 inclusion (Duncan et al., 1997). We observed a regulated intron retention at a similar position in CLK4, suggesting a similar regulation. Thus, it is possible that regulated retained introns play a role in the autoregulation of splicing factors in human brain, which could contribute to the large changes in alternative exon usage observed in AD progression. The poor conservation between species indicates that the principle of using regulated retained introns in pre-mRNAs is conserved between species, but not the actual sequences.

#### Regulated intron retention in entorhinal cortex correlates with Braak NFT stages

To investigate a possible correlation between the degree of regulated intron retention and Braak stages, we calculated intron retention as the ratio (PIR, per cent intron retention) between exon-intron-junction reads to the combined number of exon-junction and intron-junction reads for each condition. This analysis identified 1,000 Braak NFT-stage correlated introns in entorhinal and/or temporal regions, of which 171 retained introns showed enough nanopore sequencing coverage. 131 nanopore confirmed sequences showed correlation with Braak stages, corresponding to a validation rate of 76.6%. There is a clear difference in the correlation between intron retention and Braak stages, when entorhinal cortex and temporal cortex is compared. For example, 934 retained introns are correlated with Braak stages in entorhinal cortex (correlation ≤−0.8 or ≥0.8), but only ten of them correlate with Braak stages in temporal cortex Supplementary Table 8. Thus, a change in intron retention regulation correlates with the progression of Alzheimer’s disease in entorhinal cortex.

### Circular RNAs

CircRNAs are a new class of RNAs that are generated through backsplicing, i.e., the connection of a 5′ splice site with an upstream 3′ splice site (Hanan et al., 2017; Hsiao et al., 2017; Yang et al., 2022). For backsplicing to occur, the regulated splice sites need to be brought into close proximity, likely through pre-mRNA structures. In primate systems, these structures are frequently provided by primate-specific Alu elements (Jeck et al., 2013). CircRNAs are generally expressed at much lower levels than their linear counterparts, usually around 1% of the corresponding mRNA (Jeck et al., 2013; Yang et al., 2022). CircRNAs are highly expressed in the brain and often enriched at synapses. They are mostly cytosolic and more stable than linear mRNAs (Jeck et al., 2013).

The function of circRNAs is still enigmatic. circRNAs can act through sequestration of miRNAs, i.e., act as miRNA sponges (Hansen et al., 2013). Recent reports show that circRNAs can be translated through an unknown mechanism (Lukiw, 2013; Legnini et al., 2017). The translation of circRNAs was unexpected, as they lack a 5′ cap structure or other known ribosomal entry sites. We recently reported that adenosine to inosine (A > I) RNA editing strongly promotes circMAPT translation (Welden et al., 2022), suggesting that circRNAs could function as templates for protein production.

CircRNA expression in AD has previously determined in a large study that analyzed AD samples from parietal cortex (Dube et al., 2019). This study revealed 33 circRNAs that are associated with AD. When combined with publicly available datasets 14 circRNAs were associated with Braak stage (Dube et al., 2019). Candidate approaches also revealed associations between circRNA expression and AD. For example, circIGF2R is associated with AD in middle temporal cortex (Bigarre et al., 2021) and an association of circCDR1-AS with AD has been reported (Lukiw, 2013; Akhter, 2018). We thus analyzed the expression of circRNAs in entorhinal cortex.

#### Overall circular RNA expression indicates Braak stages

We determined circRNA expression in the seven entorhinal cortex samples representing Braak “0” (aged matched control) to Braak 6 that were characterized for mRNA and retained intron expression. circRNAs were sequenced after rRNA removal and RNase R treatment. We detected a total of 11,873 circular RNAs in 630 million reads (Supplementary Table 9).

**Figure 5:**
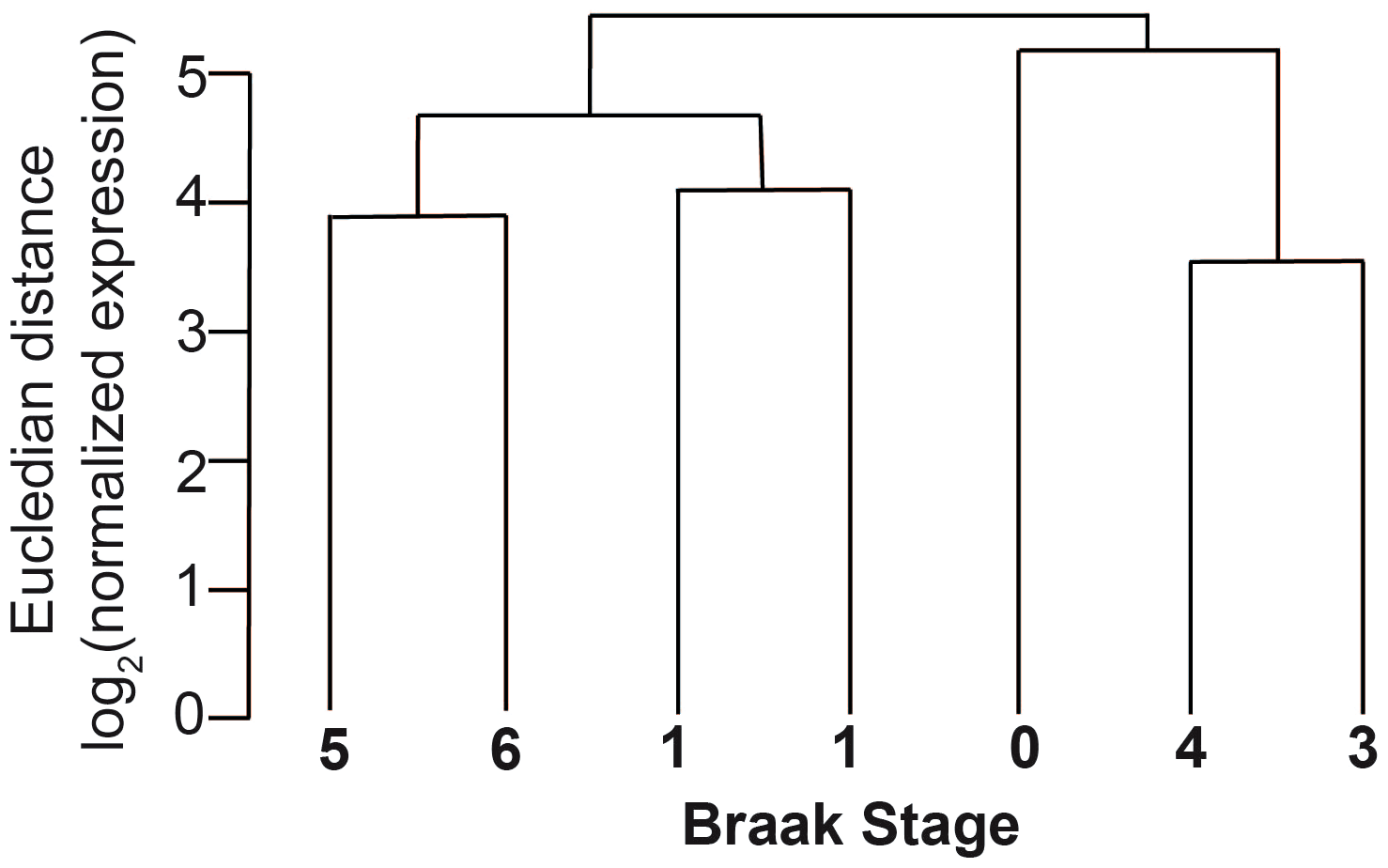
Clustering of circRNAs of different Braak stages in entorhinal cortex. The normalized Eucledian expression distance was compared with Braak NFT stages.

The sequences were first analyzed by unsupervised circRNA clustering that compared the expression levels of the circRNAs with Braak stages. We found that the samples cluster according to Braak stages, indicating that overall circRNA expression could be used as a molecular indicator for AD progression in entorhinal cortex (Figure 5). These data are in agreement with the expression of circRNAs in parietal cortex that correlated with the observed clinical dementia rating (Dube et al., 2019).

#### The expression of circular RNAs and mRNAs differ in entorhinal cortex

CircHIPK3 is among the most highly expressed circRNAs and is often used as an internal control. We therefore determined circRNAs that were expressed at a higher level than circHIPK3 by normalizing circRNA expression with CIRIquant using the linear counts for normalization (Zhang et al., 2020). We identified 21 circRNAs from all Braak stages (Table 3) that were more abundantly expressed than circHIPK3.

**Table 3:** The most abundantly expressed circRNAs in entorhinal cortex.

Backsplice junction coordinates (hg 19)	Gene symbol	Mean TMM expression	circRNA rank	linear RNA rank	Gene name	function
chrX:139865340|139,866,824	circCDR1-as	53.54	1	34,236	Cerebellar Degeneration Related Protein 1, antisense	unknown
chr1:117944808|117,963,271	MAN1A2	7.94	2	7,030	Mannosidase Alpha Class 1A Member 2	Protein glycosylation
chr6:136709531|136,710,655	MAP7	4.89	2	754	microtubule-associated protein 7	Stabilizes microtubles
chr1:16891302|16,893,846	NBPF1	4.61	2	5,529	Neuroblastoma Breakpoint Family Member 1	unknown
chr6:4891947|4,892,613	CDYL	4.59	3	7,957	chromodomain protein, Y-like	Chromodomain, chromatin binding
chr4:144464662|144,465,125	SMARCA5	3.85	3	2,774	SWI/SNF related, matrix associated, actin dependent regulator of chromatin, subfamily a, member 5 (SMARCA5)	helicase and ATPase activities to regulate chromatin
chr6:73016961|73,043,538	RIMS1	3.88	4	4,019	Regulating Synaptic Membrane Exocytosis 1	synaptic vesicle exocytosis, regulation of voltage-gated calcium channels
chr9:103261047|103,279,053	TMEFF1	3.44	5	3,844	transmembrane protein with EGF-like and two follistatin-like domains 1	Signaling during neuronal pattering
chr2:72945232|72,960,247	EXOC6B	2.85	8	4,105	exocyst complex component 6B	exocytosis
chr8:26439485|26,441,499	DPYSL2	1.97	8	230	dihydropyrimidinase-like 2	promotes microtubule assembly
chr2:40655613|40,657,444	SLC8A1	2.00	9	9,179	Solute Carrier Family 8 Member A1	calcium antiporter
chr6:73005640|73,043,538	RIMS1	1.91	9	4,019	Regulating Synaptic Membrane Exocytosis 1	synaptic vesicle exocytosis, regulation of voltage-gated calcium channels
chr4:39739040|39,776,553	UBE2K	2.26	10	2,414	ubiquitin-conjugating enzyme E2K (UBE2K)	ubiquitin-conjugating enzyme
chr3:196118684|196,129,890	UBXN7	1.95	10	9,266	UBX domain containing 7	Ubiquitin-binding adapter
chr15:64791492|64,792,365	ZNF609	1.72	10	6,937	Zinc finger protein	transcription
chr2:72958136|72,960,247	EXOC6B	1.92	12	4,105	exocyst complex component 6B	exocytosis
chr11:46098305|46,113,774	PHF21A	1.64	12	5,132	PHD Finger Protein 21A	Part of histone deacetylase complex
chr8:51351103|51,363,289	SNTG1	1.55	12	10,424	syntrophin, gamma 1	mediating gamma-enolase trafficking to the plasma membrane
chr1:211952260|211,966,532	LPGAT1	1.39	13	5,815	lysophosphatidylglycerol acyltransferase 1	acylation of lysophosphatidylglycerol to phosphatidylglycerol
chr6:136704809|136,710,655	MAP7	1.05	13	754	microtubule-associated protein 7	Stabilizes microtubles
chr16:80718435|80,719,026	CDYL2	1.34	14	12,182	chromodomain protein, Y-like 2	chromodomain
chr11:33307959|33,309,057	HIPK3	1.38	15	5,069	homeodomain interacting protein kinase 3	Serine/threonine-protein kinase transcription

The most abundantly expressed circRNA was circCDR1-as (also named ciRS-7), a circular RNA that derives from a pre-mRNA in antisense to the cerebellar degeneration-related protein 1 (CDR1) RNA. CircCDR1-as downregulates CDR1, acts as a sponge for miR-7 and is itself regulated by miR-671 (Hansen et al., 2011). circCDR1-as knockout leads to impaired sensorimotor gating in a mouse model (Piwecka et al., 2017).

Through alternative splice site usage, two pre-mRNAs, EXPOC6B and MAP7 generate more than one highly expressed circRNA. EXOC6B is part of the exocyst complex that transports secretory vesicles to the plasma membrane before vesicle-membrane fusion. MAP7 (ensconsin) is a microtubule associate protein that modulates microtubule function (Masson and Kreis, 1995; Bulinski et al., 2001). Other circular RNAs, including MAN2A1, DOCK1, and HOMER1 also generate multiple circRNA isoforms that are however not as strongly expressed (Figure 5).

The genes that strongly express circRNAs act in various pathways, among them exocytosis/neurotransmission (RIMS1, RIMS2, EXOC6B), ubiquitin pathway (UBE2K, UBXN7, ANKIB1), chromatin modeling (CDYL, CDYL2, SMARCA5, PHF21A, HIPK3), microtubule assembly (DPYSL2, MAP7) and signaling (TMEFF1, SNTG1, LPGAT1, SLC8A1), (Table 3; Supplementary Table 10).

We next determined the expression of the linear counterparts of circRNAs and found that the linear mRNAs were not as prominently expressed. For example, circMAN1A2 is the second most abundant circRNA, but the linear MAN1A2 RNA ranked 7,030 (Table 3). The data indicate that the abundance of circRNAs in entorhinal cortex is regulated differently than the abundance of linear mRNAs, suggesting that circRNAs could be new biomarkers for AD. Further, similar to HEK293 cells (Jeck et al., 2013), circHIPK3 is abundantly expressed in entorhinal cortex and can be used as a loading control.

### Numerous circRNAs correlate with Braak stages

We next investigated a possible correlation between Braak stages and circRNA expression. We compared the combined Braak 0 and 1 stages with the combined Braak 5 and 6 stages and identified 276 regulated circRNAs, whose expression correlated with the Braak stages. Regulated circRNAs were defined as circRNAs showing a fold-change larger than 1.5 with a *p*-value smaller than 0.05 (Table 4; Supplementary Table 11).

**Table 4:** Correlation of circRNAs with Braak stages in human entorhinal cortex.

	Up	Down	All
Number of regulated circRNA	80	196	276
Min fold-change	2.45	2.19	2.19
Average fold-change	23.21	24.73	24.29
Max fold-change	88.34	150.81	150.81
Gene host with the max fold change	CCDC173	HNRNPAB1	HNRNPA2B1

**Figure 6:**
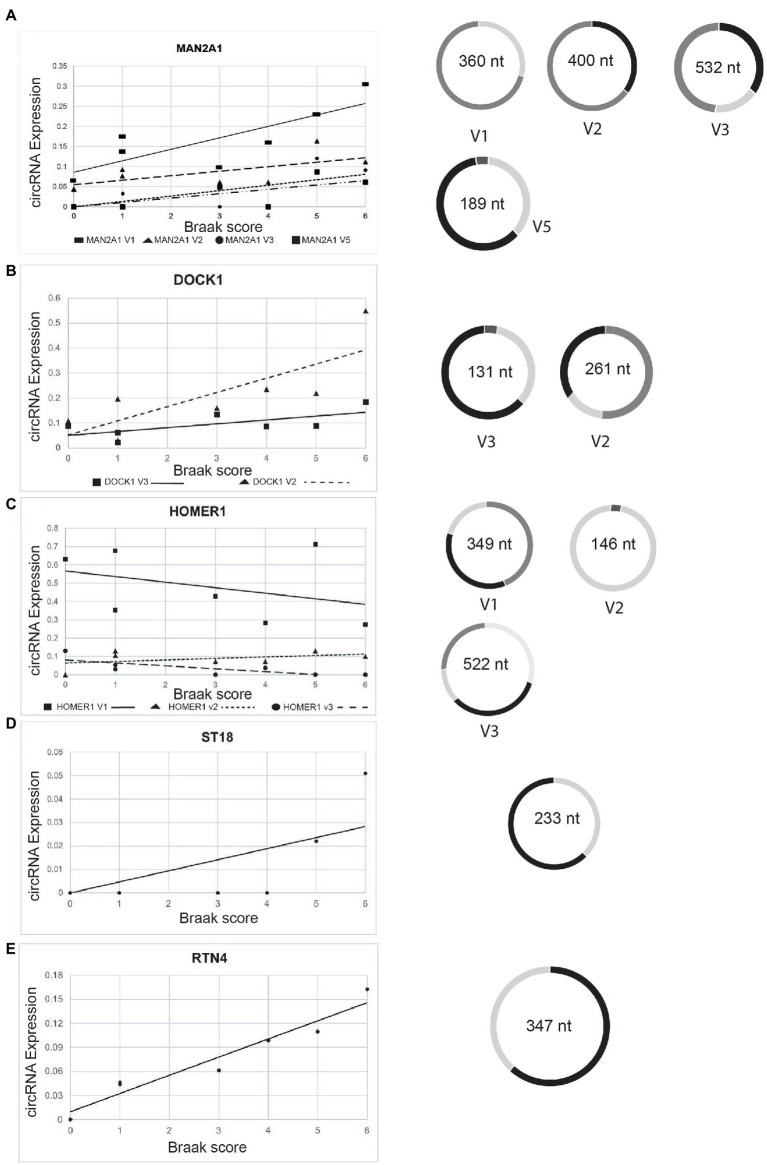
Change of expression of selected circRNAs in entorhinal cortex according to Braak stages. The relative expression levels of the genes indicated are plotted against the Braak stages. Structures of various circRNA isoforms are shown on the right. **(A)** circMAN2A1, **(B)** circDOCK1; **(C)** circHOMER1; **(D)** circST18; **(E)** circRTN4.

Importantly, five of these pre-mRNAs (DOCK1, HOMER1, MAN2A1, RTN4, and ST18) were also found to correlate with dementia severity in the parietal cortex (Dube et al., 2019), suggesting that these circRNAs in general correlate with AD progression (Figure 6). The expression of circDOCK1, circMAN2A1, and circST18 correlated with their linear counterparts (*r* = 0.885; 0.784; 0.717 respectively). However, there was no correlation for RTN4 and HOMER1 (*r* = −0.418; −0.223 respectively). Only DOCK1 mRNA expression correlates with Braak stages that did not reach statistical, significance (*r* = 0.775. *p* = 0.12). The other corresponding mRNAs did not show strong correlation with Braak stages.

We next analyzed the structure of the five circRNAs with potential predictive value in detail by mapping the internal exon junction reads. We found that DOCK1, HOMER1 and MAN2A1 pre-mRNAs generate multiple circRNAs through different exon usage. With the exception of one of the HOMER circRNAs, all circRNAs increased with the Braak stages.

In summary, a subset of circRNAs reflect Braak stages in entorhinal cortex, five of which have also been shown to correlate with disease progression in parietal cortex (Dube et al., 2019).

#### Adenosine to inosine RNA editing in circRNAs correlates with Braak stages in entorhinal cortex

We recently reported that the circRNAs from the microtubule associate protein tau (MAPT) are translated after they undergo adenosine to inosine (A > I) RNA editing, catalyzed by ADAR1 or ADAR2 (ADAR: adenosine deaminase acting on RNA; Welden et al., 2022). We thus determined the editing status of the circRNAs found in entorhinal cortex. As inosines are read as guanosines, A > I editing can be determined by identifying A > G changes in the sequence data.

**Figure 7:**
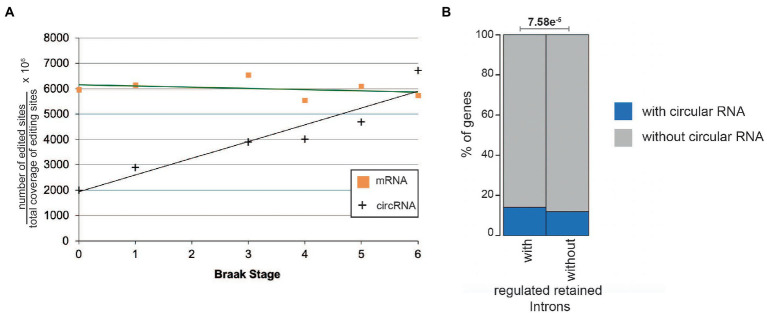
RNA editing of mRNAa and circular RNAs in the entorhinal cortex. **(A)** A > G changes in circular and linear RNAs from entorhinal cortex were calculated and normalized to the total sequence count at the individual sites. r^2^ for the circular RNAs is 0.89. **(B)** Per cent of genes expressing at least one regulated retained intron and one circular RNA (blue) or no circular RNAs (gray).

When normalized to the total amount of base pairs there is a strong correlation between Braak stages and the number of edited sites (Figure 7A). As a control for DNA variations, we performed the same calculation for linear RNAs, where no such correlation could be detected. We conclude that specifically for circRNAs the overall A > I editing increases during Alzheimer progression in entorhinal cortex, but is unchanged in linear mRNAs. Next, we asked whether the same gene expresses a regulated retained intron and a circular RNA. We found a positive correlation, namely that genes that contain a regulated retained intron are more likely to express a circular RNA (Figure 7B). However, none of the genes expressing a circRNA with a strong correlation to Alzheimer’s disease (HOMER1, DOCK1, KCNN2, ICA1, RMN1, ATRNL1, ST18, MAN2A1, EXOSC1, and RTN4) expressed a regulated retained intron.

#### A > I editing of circular RNAs promotes translation of MAN2A1

We recently reported that A > I RNA editing promotes the translation of two circMAPT RNAs and circUPF1 (Welden et al., 2022). To determine whether A > I editing also promotes translation of circRNAs deregulated in entorhinal cortex (Table 3), we investigated circMAN2A1 as it is highly expressed in entorhinal cortex and as its expression increases with Braak stages (Figure 6A). circMAN2A1 was found to correlate with AD status in parietal cortex (Dube et al., 2019).

**Figure 8:**
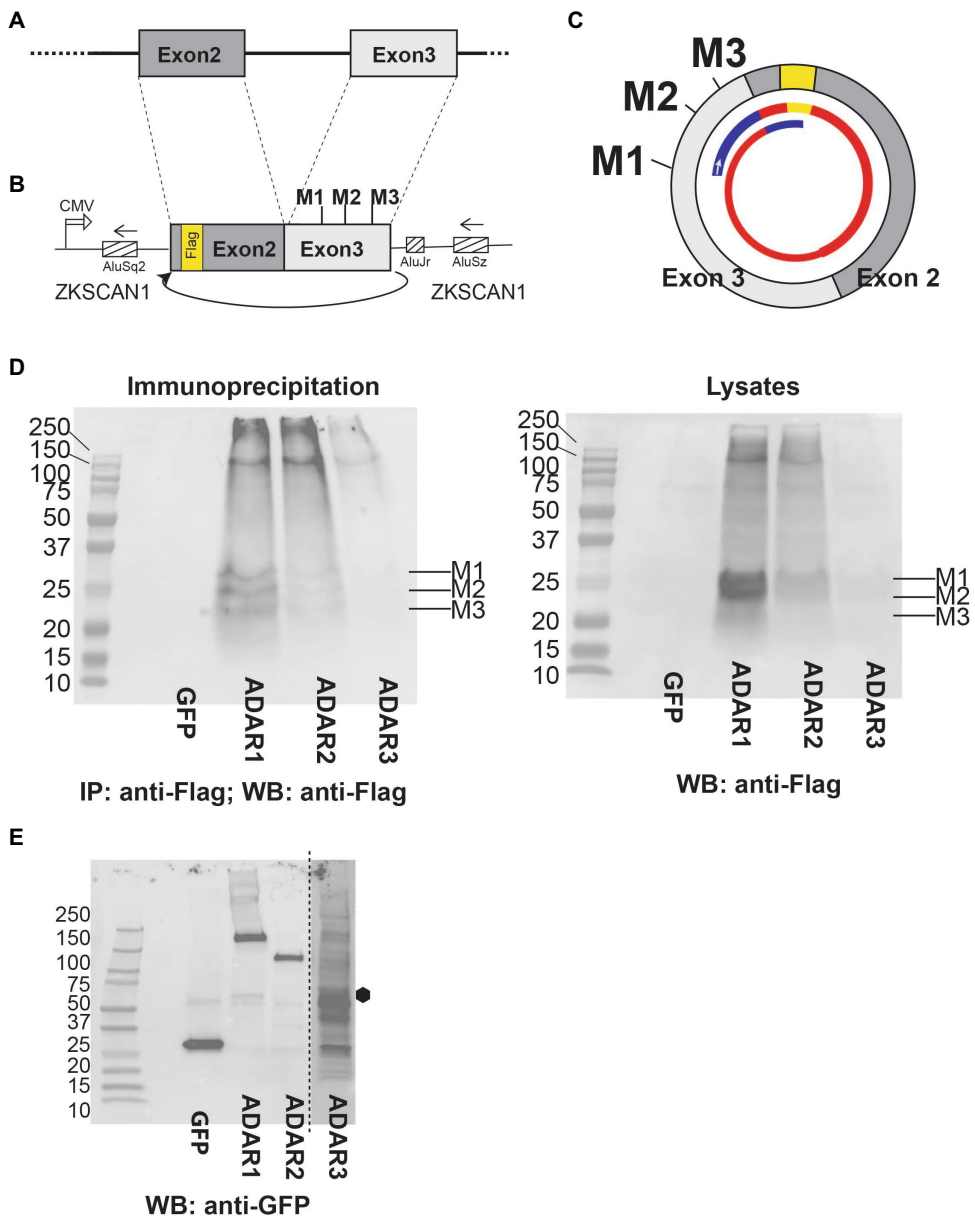
After adenosine to inosine RNA editing, circMAN2A1 is expressed as protein. **(A)** Structure of the genomic region of MAN2A1 leading to backsplicing and **(B)** structure of the expression construct that is flanked by ZKSCAN1 introns (Welden et al., 2022), a curved arrow indicates the backsplicing. **(C)** 400 nt circMAN2A1 formed by the expression construct. The three start codons M1-M3 are indicated. The inner thinner circle depicts the predicted protein. Blue: novel circMAN2A1-specific peptides, yellow: Flag tag, red: proteins shared with the linear MAN2A1 that encompass the N-terminal catalytic domain of MAN2A1 and a predicted Rho binding domain. The small arrow indicates the clockwise direction of translation. The nucleotide and amino acid sequences are in Supplementary Figure 3. **(D)** The circMAN2A1 expression construct was cotransfected with expression clones for GFP, ADAR1, ADAR2 and ADAR3 in HEK293 cells. Left: The immunoprecipitation and detection was with anti-Flag antibody. Right: Western blot of the cell lysates. **(E)** Detection of ADAR overexpression in the cell lysates. Lysates were analyzed in Western Blot using anti-GFP. All constructs were GFP-tagged, ADAR3 exposure is 4× the rest of the blot, the expected size is indicated with a hexagon.

We cloned the genomic region lacking the internal intron under the control of a CMV promoter, flanked by ZKSCAN1 Alu-elements. For detection of proteins, we introduced a 3x Flag tag in the cDNA (Figures 8A,B). Importantly, there are no in-frame start codons upstream of the Flag tag in the ZKSCAN1 intronic region. CircMAN2A1 contains three AUG start codons M1-M3 that are in frame with the Flag tag. There are seven other AUG triplets that are out of frame with the Flag tag (Supplementary Figure 3A). The expression construct was cotransfected with GFP, ADAR1, 2 and 3 expression constructs into HEK293 cells. The protein was immunoprecipitated using anti-Flag and detected using anti-Flag. Similar to the circMAPT system (Welden et al., 2022), we detected protein expression in the presence of ADAR1 or 2 (Figure 8D). No expression was detected when we used the catalytically inactive variant ADAR3 or cotransfected GFP as a control. In the immunoprecipitates we observed three bands that corresponded to the predicted sizes of 21.2 kDa, 21.8 kDa, and 22.5 kDa (Supplementary Figures 3B,C), marked M1-M3 in Figure 8D. The top two bands run 3–4 kDa higher than expected, possibly due to posttranslational modification. The expression of the protein appears relatively high, as expression from M1 and M2 can be detected in the crude lysates in Western Blot as well (Figure 8D). The overexpression of ADAR1-3 was confirmed by Western blot using their GFP tag. We observed a weak expression and partial degradation of the catalytic inactive variant ADAR3 (Figure 8E).

These data indicate that similar to circMAPT and circUPF1 (Welden et al., 2022), circMAN2A1 can be translated after A > I RNA editing. A possible molecular mechanism could be an inosine-recognizing RNA binding protein that mediates an interaction between the edited circRNA and the ribosome.

CircMAN2A1 thus could act as protein. The protein will contain sequences that are not predicted from the linear mRNA (blue lines Figure 8C). It also encompasses part of the N-terminal catalytic domain of the linear MAN2A1 and a predicted Rho binding domain. It is possible that the expression levels of circRNA-encoded proteins increase with progressive Braak stages, as the A > I editing of circular RNAs generally increases during Alzheimer’s disease progression (Figure 7).

## Discussion

### Changes in mRNA expression during AD progression

We used a small number of samples representing AD progression from Braak stage I to VI. We concentrated on entorhinal and temporal cortex. Similar to previous studies we found widespread changes in mRNA expression. Using RNA markers for different cell types, we also demonstrated changes in cell composition, most notably reflecting the loss of neurons. A novel finding was the widespread changes in alternative splicing that correlated more strongly with Braak NFT stages than with changes in mRNA expression. We did not detect any evidence of trans-splicing, which has for example been reported to occur between ERBIN and SMN (Ottesen et al., 2019).

### Regulated retained introns

As we observed a strong correlation between alternative exon usage and Braak NFT stages, we determined changes in regulated retained introns. It has been shown in mouse models that numerous neuronal polyadenylated nuclear transcripts exist that contain only one intron (Mauger et al., 2016). This arrangement allows regulated removal of the retained intron facilitating a fast response of gene expression to a stimulus.

Similar retained introns have been detected in rat spinal cord, where they are deregulated during spinal cord injury (Hart et al., 2022). The regulated retained introns in human, mouse and rat are dispersed throughout the coding sequence of the mRNA. As the introns contain stop codons in all three frames, this prevents expression of protein even if the intron-containing mRNAs are exported from the nucleus to the cytosol. This indicates that the sequence of splicing events within a pre-mRNA evolved differently in different organisms. Consequently, the nature of the last intron to be removed in response to the stimulus could be different in different organisms. This mechanism is affected during Alzheimer’s disease progression. There is almost no conservation of regulated intron retention between human, mouse and rat. This indicates that the principle of a ‘pre-spliced’, polyadenylated mRNA is conserved, but different introns have evolved in various species to allow pre-mRNAs to rapidly respond to a stimulus. Notable in AD progression is the strong deregulation of retained introns in entorhinal cortex that is far less pronounced in temporal cortex, but the molecular reasons for this difference remain to be determined.

### CircRNAs can act as proteins

Using the same tissue as in the prior analyses described above, we determined the changes in circRNA expression. We found a widespread deregulation of circRNAs that showed a correlation with Braak NFT stages stronger than alternative exon usage of mRNA expression. Genes previously implicated in AD, such as *TREM2, CR1, SHIP1, BIN1, CD33, CLU, PCLG2* (Gale et al., 2014; Karch and Goate, 2015) do not generally strongly express circRNAs. The only exception is *PICALM* that generates several circPICALM isoforms. The difference between linear and circRNAs in their correlation with AD progression suggests that they could act through different molecular mechanisms.

We found that five of the ten circRNAs that were associated with cognitive decline in parietal cortex (Dube et al., 2019) also change in entorhinal cortex. Importantly, the circRNAs were differently regulated than their linear counterparts. For example, the second most abundant circRNA (and the most abundant circRNA associated with a coding transcript) was *MAN1A2*, whereas linear *MAN1A2* is ranked as number 7,030. circMAN2A1, circDOCK1, circHOMER1, and circST18 were among the 50-most expressed circular RNAs, but their mRNA counterparts rank between numbers 4,000 and 9,000 in terms of expression. An exception is circRTN4 which is among the 50-most expressed circular RNAs similar to linear RTN4 that ranks between 100–180. It is thus possible that there is a specific molecular regulation that increased expression of a subset of circular RNAs.

We previously found that adenosine to inosine (A > I) RNA editing strongly promotes translation of circMAPT and circUPF1. We therefore tested a change in A > I editing in all circRNAs from entorhinal cortex and found a very strong correlation between Braak stages and A > I editing that increases during AD progression. To further test a possible translation, we cotransfected expression clones for MAN2A1 with ADAR1-3 expressing clones and again found A > I dependent circMAN2A1 protein expression. circMAN2A1 spans 400 nt, i.e., a rolling circle translation will generate a frameshift, which is different from the circMAPT RNAs whose numbers are divisible by three. We observed three protein bands, likely due to initiation from three start codons that are out of frame with the ‘linear MAN2A1” ORF and not used as methionines in the linear MAN2A1 mRNA. The circMAN2A1 protein starts in a “circular frame” in exon 3. Upon entering exon 2, the linear MAN2A1 frame is used. This frame is used in the rolling circle translation when translation proceeds from exon 2 to exon 3, as in the linear MAN2A1. However, when translation proceeds from exon 3 back to exon 2, the ‘circular frame’ is used, and a stop codon is encountered (Figure 8C; Supplementary Figure 3B). Thus, translation of circMAN2A1 generates a protein containing N and C-terminal circRNA-specific sequences that flank part of the linear MAN2A1 protein. This linear protein part contains the N-terminal part of the catalytic domain. The function and possible catalytic properties of the protein remain to be determined.

**Figure 9:**
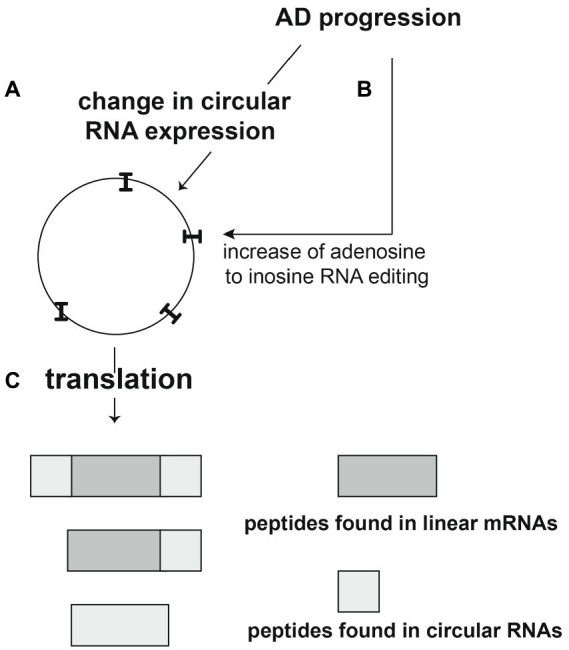
Proposed role of circular RNAs in Alzheimer’s disease. **(A,B)** AD progression leads to an increase of several circRNAs and generally to an increase of Adenosine to inosine RNA editing of these circRNAs (indicated as “I”). **(C)** The RNA editing leads to translation of the circRNAs. circRNAs can use reading frames different from linear RNAs, leading to the translation of novel peptides that could be Alzheimer-specific and could contribute to inflammation during AD progression.

During AD progression there is a strong overall increase in A > I editing per circular RNA, suggesting that circRNA translation could be elevated in later stages of AD (Figure 9). These circRNA derived proteins will contain novel peptides that could contribute to the inflammatory response seen in AD and could encode possibly new catalytic functions. Thus, deregulation and translation of circRNAs could be a decisive contributor to AD.

## Data availability statement

The datasets presented in this study can be found in online repositories. The circMAN2A1-400 expression clone was deposited to Addgene, ID198837. https://www.addgene.org/198837/.

## Ethics statement

All patients and/or their next of kin provided consent forms and all human research was performed in accordance with the U. Kentucky IRB.

## Author contributions

KAM, JRW, and GM performed cloning and transfection analysis. SS and SH performed RNA isolation and nanopore sequencing. AH performed sequencing. NR and PG did the bioinformatic analysis. PN and SS planned and oversaw the experiments. All authors contributed to the article and approved the submitted version.

## Funding

This work was supported by R01 AG061111 (PN), R01 AG057187 (PN), P30 AG072946 (PN), RF1 NS118584 (PN), and U.S. Department of Defense AZ180075 (SS); and National Institute on Aging R21AG064626 (SS) and NSF MCB-2221921 (SS).

## Conflict of interest

PG and NR were employed by Genosplice.

The remaining authors declare that the research was conducted in the absence of any commercial or financial relationships that could be construed as a potential conflict of interest.

## Publisher’s note

All claims expressed in this article are solely those of the authors and do not necessarily represent those of their affiliated organizations, or those of the publisher, the editors and the reviewers. Any product that may be evaluated in this article, or claim that may be made by its manufacturer, is not guaranteed or endorsed by the publisher.
