# Supplementary figures and images for: Alzheimer’s disease pathogenetic progression is associated with changes in regulated retained introns and editing of circular RNAs
Source: Front Mol Neurosci. 2023 May 5;16:1141079. doi: 10.3389/fnmol.2023.1141079 (PMC10231643; doi:10.3389/fnmol.2023.1141079)

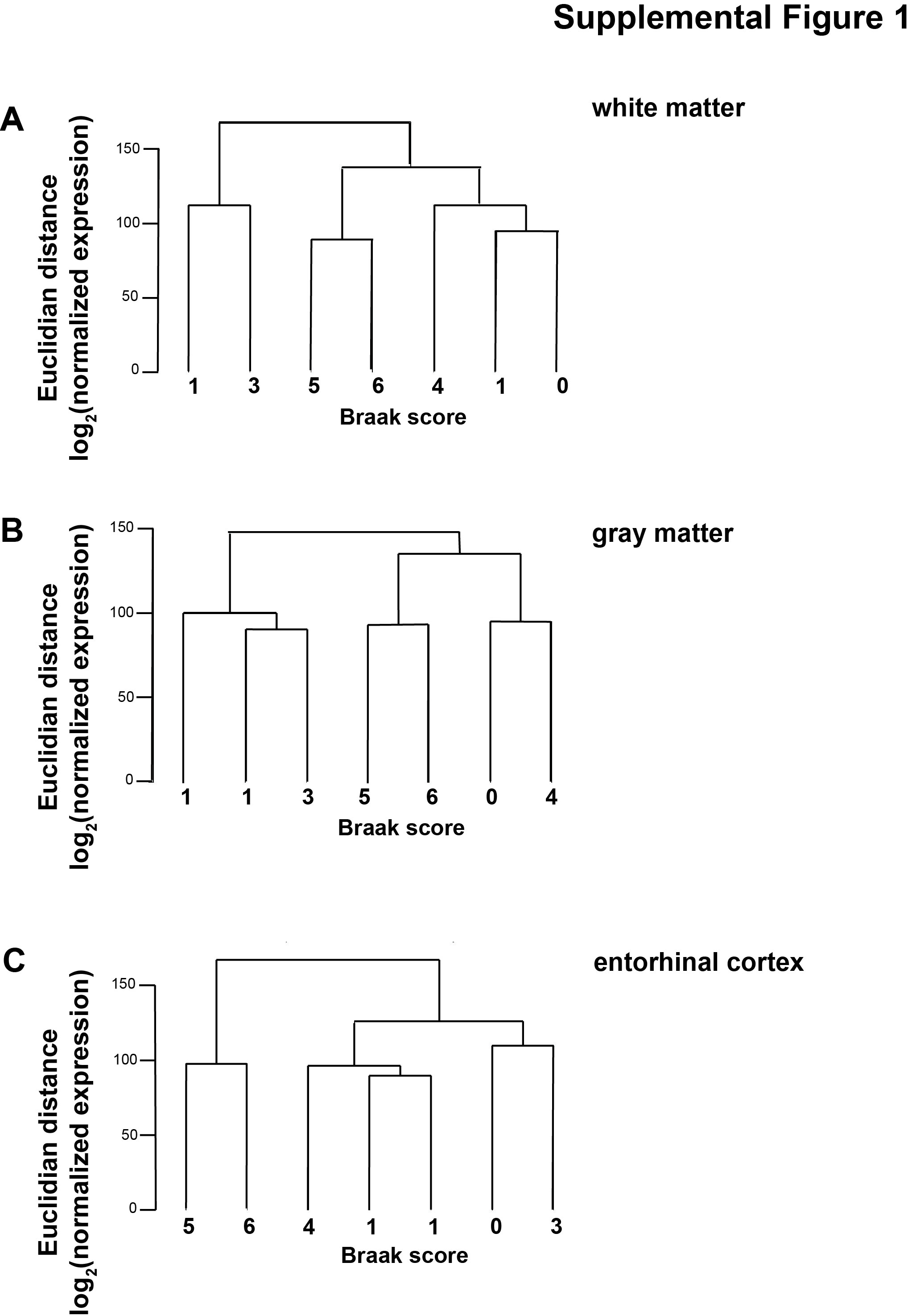

Supplement: Supplementary file 12 [file Image_1.jpeg]

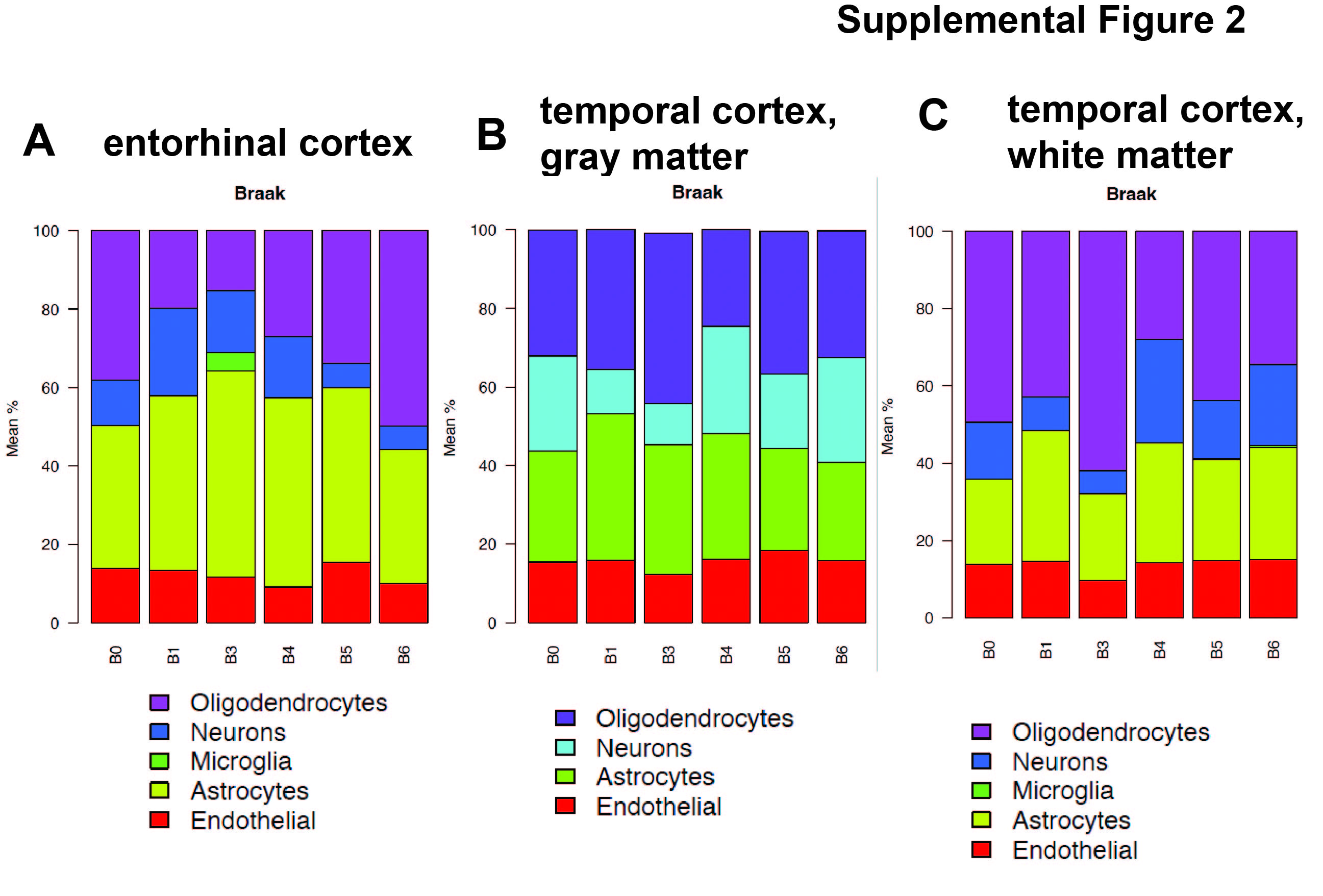

Supplement: Supplementary file 13 [file Image_2.jpeg]
