# Supplementary material for: Alzheimer’s disease pathogenetic progression is associated with changes in regulated retained introns and editing of circular RNAs
Source: Front Mol Neurosci. 2023 May 5;16:1141079. doi: 10.3389/fnmol.2023.1141079 (PMC10231643; doi:10.3389/fnmol.2023.1141079)
Supplement: Supplementary file 14 [file Data_Sheet_1.PDF]

circMAN2A1-400 sequence and predicted peptide sequences

A. Sequence of the expression cassette

Exon2, Exon 3, 3x Flag tag

```
GGCCAGCTCTCAgactacaaggaccacgacggtgattacaaggatcacgacatcgattacaagga
cgacgacgacaagATGTTGCAAGAAAAAATAGACCATTTGGAGCGTTTGCTAGCTGAGAATAATGAGAT
CATCTCAAATATTAGAGACTCAGTCATCAATTTGAGTGAGTCTGTGGAGGATGGTCCGAAAAGTTCACAAA
GCAATTTCAGCCAAGGTGCTGGCTCACATCTTCTGCCCTCACAATTATCCCTCTCAGTTGACACTGCAGAC
TGTCTGTTTGCTTCACAAAGTGGAAGTCACAATTCAGATGTGCAGATGTTGGATGTTTACAGTCTAATTTC
TTTTGACAATCCAGATGGTGGAGTTTGGAAGCAAGGATTTGACATTACTTATGAATCTAATGAATGGGACA
CTGAACCCCTTCAAGTCTTTGTGGTGCCTCATTCCCATAACGACCCAG
```

B. Translation of open reading frames containg the FLAG tag

```
atg gtg gag ttt gga agc aag gat ttg aca tta ctt atg aat cta atg aat ggg aca ctg
 M   V   E   F   G   S   K   D   L   T   L   L   M   N   L   M   N   G   T   L
aac ccc ttc aag tct ttg tgg tgc ctc att ccc ata acg acc cag ggc cag ctc tca gac
 N   P   F   K   S   L   W   C   L   I   P   I   T   T   Q   G   Q   L   S   D
tac aag gac cac gac ggt gat tac aag gat cac gac atc gat tac aag gac gac gac gac
 Y   K   D   H   D   G   D   Y   K   D   H   D   I   D   Y   K   D   D   D   D
aag atg ttg caa gaa aaa ata gac cat ttg gag cgt ttg cta gct gag aat aat gag atc
 K   M   L   Q   E   K   I   D   H   L   E   R   L   L   A   E   N   N   E   I
atc tca aat att aga gac tca gtc atc aat ttg agt gag tct gtg gag gat ggt ccg aaa
 I   S   N   I   R   D   S   V   I   N   L   S   E   S   V   E   D   G   P   K
agt tca caa agc aat ttc agc caa ggt gct ggc tca cat ctt ctg ccc tca caa tta tcc
 S   S   Q   S   N   F   S   Q   G   A   G   S   H   L   L   P   S   Q   L   S
ctc tca gtt gac act gca gac tgt ctg ttt gct tca caa agt gga agt cac aat tca gat
 L   S   V   D   T   A   D   C   L   F   A   S   Q   S   G   S   H   N   S   D
gtg cag atg ttg gat gtt tac agt cta att tct ttt gac aat cca gat ggt gga gtt tgg
 V   Q   M   L   D   V   Y   S   L   I   S   F   D   N   P   D   G   G   V   W
aag caa gga ttt gac att act tat gaa tct aat gaa tgg gac act gaa ccc ctt caa gtc
 K   Q   G   F   D   I   T   Y   E   S   N   E   W   D   T   E   P   L   Q   V
ttt gtg gtg cct cat tcc cat aac gac cca ggg cca gct ctc aga cta caa gga cca cga
 F   V   V   P   H   S   H   N   D   P   G   P   A   L   R   L   Q   G   P   R
cgg tga tta caa gga tca cga cat cga tta caa gga cga cga cga caa gat gtt gca aga
 R   -
```

C. Predicted protein

MVEFGSKDLTLLMNLMNGTLNPFKSLWCLIPITTQGQLSDYKDHDGDYKDHDIDYKDDD
DKMLQEKIDHLERLLAENNEIISNIRDSVINLSESVEDGPKSSQSNFSQGAGSHLLPSQ
LSLSVDTADCLFASQSGSHNSDVQMLDVYSLISFDNPDGGVWKQGFDITYESNEWDTEP
LQVFVVPHSHNDPGPALRLQGPRR-

yellow flag tag, blue circMAN2A1-specific peptide, Red MAN2A1 linear

Molecular weight after initiation at red, green and blue start codon

Mwt 22,515.82
Mwt 21,181.23
Mwt 20,823
